# Differential proteomic analysis and pathogenic effects of outer membrane vesicles derived from *Acinetobacter baumannii* under normoxia and hypoxia

**DOI:** 10.1371/journal.pone.0283109

**Published:** 2023-03-15

**Authors:** Sachio Suzuki, Phawinee Subsomwong, Kouji Narita, Noriaki Kawai, Takahito Ishiai, Wei Teng, Rojana Sukchawalit, Akio Nakane, Sadatomo Tasaka, Krisana Asano

**Affiliations:** 1 Department of Respiratory Medicine, Hirosaki University Graduate School of Medicine, Hirosaki, Aomori, Japan; 2 Department of Microbiology and Immunology, Hirosaki University Graduate School of Medicine, Hirosaki, Aomori, Japan; 3 Institute for Animal Experimentation, Hirosaki University Graduate School of Medicine, Hirosaki, Aomori, Japan; 4 Laboratory of Biotechnology, Chulabhorn Research Institute, Lak Si, Bangkok, Thailand; 5 Department of Biopolymer and Health Science, Hirosaki University Graduate School of Medicine, Hirosaki, Aomori, Japan; University of Cambridge, UNITED KINGDOM

## Abstract

*Acinetobacter baumannii* is a major causative agent of nosocomial infections and its outer membrane vesicles (AbOMVs) have been shown to be involved in pathogenicity by transporting virulence factors and transferring information for communication between pathogens and host cells. Despite the fact that the infected sites of *A*. *baumannii* such as lungs and skin soft tissues are hypoxic, most studies on AbOMV virulence have used AbOMVs prepared under aerobic conditions. The present study aims to elucidate the protein profile and pathogenic impact of AbOMVs released under hypoxic condition. AbOMVs were isolated from *A*. *baumannii* under normoxic and hypoxic conditions, and their protein profiles were compared. The different effects of both normoxic and hypoxic AbOMVs in cytokine response from mouse macrophages, cytotoxicity to the human lung epithelial cells, and bacterial invasion were then investigated. Our results showed that *A*. *baumannii* under hypoxia released larger amounts of OMVs with different protein profiles. Although the cytotoxic effect of AbOMVs from normoxia and hypoxia were comparable, AbOMVs from normoxia induced higher TNF-α production and invasion of *Staphylococcus aureus* and *Pseudomonas aeruginosa* than those from hypoxia. On the other hand, AbOMVs significantly enhanced *A*. *baumannii* invasion into lung epithelial cells in a dose-dependent manner. These results clearly demonstrate that AbOMVs released from normoxic and hypoxic have different impacts in pathogenesis. This finding provides new insight into the complex interactions between *A*. *baumannii*, coinfecting pathogens and host cells via OMVs, in particular the different pathogenic effects of AbOMVs under normoxic and hypoxic conditions.

## Introduction

*Acinetobacter baumannii* is an aerobic Gram-negative coccobacillus that is one of the causative agents of nosocomial infections [1]. It causes ventilator-associated pneumonia, blood stream infections, urinary tract infections, meningitis, and wound infections [2]. In recent years, the emergence of multidrug-resistant *A*. *baumannii* strains which are more difficult to treat than antibiotic-susceptible species has become one critical priority pathogen, and the World Health Organization is focusing on this issue [3–5].

Outer membrane vesicles (OMVs) released from Gram-negative bacteria have attracted attention as a virulence factor. The OMVs are generated by budding off from the outer membrane and are spherical in shape, 20–200 nm in diameter, surrounded by a lipid bilayer. These vesicles are composed of lipopolysaccharides (LPS), membrane proteins, lipids, cilia, cytosolic proteins, DNA, and RNA [6, 7]. The ligands on the membrane surface can direct OMVs to bind multiple targets. Therefore, it has been suggested that the functions of OMVs include the transport of virulence factors such as outer membrane proteins, phospholipases and lipopolysaccharide, information transfer and communication between pathogens and host cells [8, 9]. In addition, OMVs are considered to be stable vehicles because the enclosed structure of OMVs is able to protect the functional genes, DNA, RNA, proteins and other substances from degrading enzymes such as DNases, RNases and proteases [10].

The pathogenicity of *A*. *baumannii* during infectious process is associated with adhesion and invasion into the host cells, biofilm formation, induction of host cell death, and stimulation of host immune response [11, 12]. A major virulence factor is outer membrane protein A (OmpA), which is associated with adhesion, invasion, and host cell apoptosis [13]. Since OMVs can deliver the virulence factors to host cells, there have been several reports on the presence of virulence factors including OmpA in AbOMVs and their pathogenicity such as host cell death and immune responses [14]. However, most studies on AbOMV virulence have used AbOMVs prepared from aerobic conditions [15], despite the fact that the infected sites such as lungs and skin soft tissues are hypoxic [16]. Importantly, there are no reports on the amount or pathogenicity of AbOMVs prepared from hypoxic conditions, even though other bacteria such as *Pseudomonas aeruginosa* has been shown to produce large amount of OMVs when cultured under stress conditions such as hypoxia [17, 18].

The skin and lungs are the most common sites of *A*. *baumannii* infection in which other bacteria such as *Staphylococcus aureus* and *P*. *aeruginosa* often coexist. The polymicrobial infections by these pathogens are associated with severe prognosis of the diseases. One reason for this may be that the antibiotics are less effective. Carbapenem-resistant *A*. *baumannii* has been reported to protect other Gram-negative bacteria from exposure to β-lactam antibiotics [19]. It has also been reported that carbapenem-resistant *A*. *baumannii* enhanced carbapenem resistance in carbapenem-susceptible *S*. *aureus* [20]. Thus, these bacteria are thought to interact with each other and increase virulence as well as tolerance. However, the direct effect of AbOMVs on virulence of these coexisting pathogens has not been reported.

In this study, we investigated whether there is difference in the production and protein profiles of AbOMVs prepared under hypoxic condition (hAbOMVs) and under normoxic condition (nAbOMVs). In addition to the production and proteomic analysis, the pathogenic effects of hAbOMVs and nAbOMVs on host immune response, cytotoxicity, and *A*. *baumannii* invasion were compared. Moreover, the effects of these AbOMVs on the invasion of coexisting pathogens, *S*. *aureus* and *P*. *aeruginosa*, were also examined.

## Materials and methods

### Bacterial strains and growth conditions

*A*. *baumannii* ATCC19606, *S*. *aureus* ATCC1718, and *P*. *aeruginosa* ATCC15692 were used in this study. They were cultured at 37°C in tryptic soy broth (TSB; BD Bioscience, Sparks, MD). For purification of AbOMVs, *A*. *baumannii* was precultured for 16 h in TSB and used to inoculate into 4.8 L of Brain Heart Infusion medium (BD Bioscience) with 0.5% inoculum. The cultures were then placed under normoxic and hypoxic conditions for nAbOMVs and hAbOMVs, respectively. For normoxic condition, the cultures (400 mL medium in 1 L baffled flasks) were shaken at 125 rpm for 24 h, whereas for hypoxic condition, the cultures (400 mL medium in 500 mL normal conical flasks) were placed under static condition for 48 h. Thereafter, the supernatant was collected by centrifugation twice at 3,800 ×*g*, 4°C for 60 min, filtrated through 0.45 μm filter (Nalgene Rapid-Flow Filters, Thermo Fisher Scientific, Waltham, MA) to remove remaining bacterial cells and kept -80°C until AbOMVs purification. It should be noted that the oxygen concentration in uninoculated medium was measured using a JPB-70A dissolved oxygen analyzer (Shen Zhen Yage Technology, Guang Dong, China) [21]. The oxygen concentration for normoxia and hypoxia was 6.5 mg/L and 1.0 mg/L, respectively.

For bacterial infection assay, *A*. *baumannii*, *S*. *aureus*, and *P*. *aeruginosa* were precultured for 16 h and inoculated into 20 mL of TSB. The cultures were incubated at 37°C under aerobic condition by shaking at 125 rpm for 5 h. The bacterial cells were collected, washed twice with phosphate-buffered saline (PBS), and adjusted to an appropriate concentration by calculating via conversion of optical density at 600 nm.

### Cells and culture conditions

Mouse macrophage RAW264.7 cells were cultured at 37°C under 5% CO_2_ in Dulbecco’s Modified Eagle Medium (DMEM; Nissui Pharmaceutical Co., Tokyo, Japan), supplemented with 10% Fetal Calf Serum (FCS; JRH Biosciences, Lenexa, KS), 0.03% L-glutamine (FUJIFILM Wako Pure Chemical Industries, Osaka, Japan), and antibiotic-antifungal combination agent (Gibco^™^ Antibiotic-Antimycotic; Thermo Fisher Scientific, Waltham, MA). Human alveolar basal epithelial A549 cells were cultures at 37°C under 5% CO_2_ in Ham’s F-12K medium (FUJIFILM Wako Pure Chemical Corporation), supplemented with 10% FCS, and antibiotic-antifungal combination agent.

### Purification of AbOMVs

AbOMVs in the culture supernatant of *A*. *baumannii* were harvested by ultracentrifugation at 128,400 ×*g*, 4°C for 90 min using a Himac CP80NX Preparative Ultracentrifuge (HITACHI, Tokyo, Japan). After removal of the supernatant, crude pellet containing AbOMVs was then washed and suspended in 2 mL of PBS. AbOMVs were further purified using an OptiPrep^™^ density gradient according to the standard protocol [22] with some modifications. Briefly, a discontinuous iodixanol gradient [45%, 35%, 30%, 25% and 15% (w/v) iodixanol solutions (OptiPrep^™^; Sigma Aldrich, St. Louis, MO) in 0.25 M sucrose/10 mM Tris, pH 7.5] was prepared. A 320 μL volume of crude-AbOMVs suspension was overlaid on the discontinuous iodixanol gradient and ultracentrifuged for 16 h at 100,300 ×*g*, 4°C. Thereafter, six fractions (720 μL each) were collected from the top of the gradient. Then, each fraction was diluted in PBS, and again harvested by ultracentrifugation at 99,800 ×*g*, 4°C for 3 h. The pellet was washed once and resuspended in an appropriate volume of PBS. Protein concentration of each fraction was measured by Bradford protein assay using Bio-Rad Protein Dye Reagent Concentrate (Bio-Rad Laboratories, Inc., Hercules, CA). The AbOMVs containing fraction was analyzed under transmission electron microscope (JEM-1230, JEOL, Tokyo, Japan) with negative staining (TI blue, Nisshin EM Co. Ltd, Tokyo, Japan). The particle concentration and size distribution of AbOMVs were analyzed using qNano instrument (Izon Science, Oxford, United Kingdom) according to the manufacture’s instruction. Briefly, the purified AbOMVs were diluted in Measurement Electrolytes (qNano reagent kit) containing 0.03% Tween 20 (FUJIFILM Wako Pure Chemical Corporation) and analyzed using NP200 nanopore membranes in comparison with 200-nm calibration particles by running at 0.62 V. Izon Control Suite software version 3.3.2.2001 was used for data analysis.

### Proteomic analysis of AbOMVs

Quantitative proteomic analyses of nAbOMVs and hAbOMVs were performed in triplicate by liquid chromatography-tandem mass spectrometry as described previously [23]. Briefly, the acetone-precipitated proteins of AbOMVs were denatured with 50% trifluoroethanol and reduced with 4 mM dithiothreitol. Free cysteine residues were alkylated prior to trypsinization. The peptides were desalted and separated using liquid chromatography. The mass spectrometer was set in an information-dependent acquisition mode. Acquired spectra were searched against the *A*. *baumannii* ATCC19606 proteins (NCBI Genbank: CP045110.1). Positive identification was considered when identified proteins and peptides reached a 1% local false discovery rate. The proteomic data obtained in this study are available using accession number PXD037014 and JPST001865 for Proteome Xchang and jPOST Repository, respectively. Differential proteomics between nAbOMVs and hAbOMVs was then analyzed. Compute pI/Mw tool-ExPASy (https://web.expasy.org/compute_pi/), PsortB v.3.0 (https://www.psort.org/psortb/) were used for predicting molecular weight and subcellular localization of each identified protein, respectively.

### Cytokine assay

RAW264.7 cells (1 × 10^6^ cells/mL/well) were seeded in 24-well culture plates and stimulated with 12.5 μg/mL AbOMVs for 48 h. The culture supernatant was then collected by centrifugation twice at 5,000 ×*g*, 4°C for 10 min to remove the residual cells. The titers of TNF-α, IL-6, and IL-10 were measured using Mouse ELISA kits (Invitrogen, Carlsbad, CA) according to the manufacturer’s instructions.

### Cytotoxic effect of AbOMVs

A549 cells (1 × 10^4^ cells/100 μL/well) were seeded in 96-well plates and incubated with 0, 1, 5, 10, 25 and 50 μg/mL AbOMVs at 37°C under 5% CO_2_. At 12 h of incubation, A549 cells were washed once with Hanks’ Balanced Salt Solution (HANKS; Nissui Pharmaceutical Co.) to remove AbOMVs. Cells were then maintained in 100 μL medium prior to cell viability assay. WST-1 Cell Proliferation Reagent (Sigma-Aldrich) was used for determining cell viability was determined using according to the manufacturer’s instruction. Briefly, WST-1 reagent (10 μL) was added into each well directly and incubated at 37°C under 5% CO_2_. After color development, the optical density at 450 nm was measured using a microplate reader (MULTISKAN Sky, Thermo Fisher Scientific). The absorbance values were subtracted with background controls (medium without A549 cells containing 0, 1, 5, 10, 25 and 50 μg/mL AbOMVs, respectively). The viability of A549 cells without AbOMVs is referred to 100%.

### Bacterial infection assay

A549 cells (5 × 10^4^ cells/mL/well) were seeded in 24-well plates and incubated at 37°C, under 5% CO_2_. At 48 h after incubation, A549 cells were washed and infected with *A*. *baumannii*, *S*. *aureus*, or *P*. *aeruginosa* at MOI = 50 in the presence of AbOMVs. The infection time was 5 h for *A*. *baumannii*, and 2 h for *S*. *aureus* and *P*. *aeruginosa*. Afterwards, the bacterial cells were removed, and the cells were washed twice with HANKS solution and once with PBS. To eliminate all extracellular *A*. *baumannii* and *S*. *aureus*, the A549 cells were treated with 0.2% Lysostaphin (FUJIFILM Wako Pure Chemical Corporation). On the other hand, the A549 cells were treated with 120 μg/mL of gentamicin (Wako Pure Chemical Corporation) to eliminate the extracellular *P*. *aeruginosa*. At 1 h of antibiotic treatment, the cells were then washed with HANKS solution and once with PBS again. To enumerate the invading *A*. *baumannii*, the A549 cells were lysed with 0.2% (3-[(3-Cholamidopropyl)-dimethylammonio]-1-propanesulfonate (CHAPS; DOJINDO, Kumamoto, Japan) for 1 h. To enumerate the invading *S*. *aureus* and *P*. *aeruginosa*, the A549 cells were lysed with 1% CHAPS for 15 min. Then, the bacterial cell suspensions were diluted and plated on tryptic soy agars. The colonies were counted at 24 h after incubation.

### Statistical analysis

Statistical differences were analyzed using the method mentioned in each figure legend. A *P*-value less than 0.05 is considered statistically significant.

## Results

### Purification of nAbOMVs and hAbOMVs

**Fig 1:**
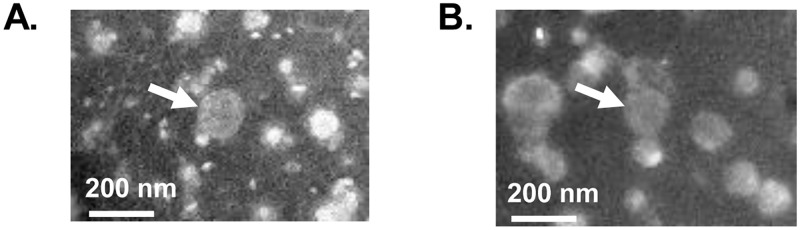
Negative-staining transmission electron micrographs of (A) nAbOMVs and (B) hAbOMVs. *A*. *baumannii* ATCC19606 were cultured under normoxic and hypoxic conditions for 24 h and 48 h, respectively. After removal of bacterial cells, nAbOMVs and hAbOMVs in the culture supernatant from normoxic and hypoxic conditions were collected and purified by step-gradient ultracentrifugation. AbOMVs (white arrows) from both conditions were detected in fraction F2.

To prepare nAbOMVs and hAbOMVs, *A*. *baumannii* was cultured under aerobic and static condition, respectively. The growth of *A*. *baumannii* under aerobic condition reached an OD_600nm_ of about 2.0 after 24 h of incubation, and that under static condition reached an OD_600nm_ of about 1.0 after 48 h. The culture supernatants from both conditions were then collected and subsequently purified by step-gradient ultracentrifugation. From six separated fractions (F1 to F6), the purified AbOMVs from each culture condition were detected in F2 as confirmed by negative-staining transmission electron microscopy (Fig 1A and 1B). The particles with an enclosed structure of approximately 200 nm in diameter were observed. Nanoparticle tracking analysis revealed that the average particle sizes of nAbOMVs and hAbOMVs were very similar, with 187 nm and 189 nm, respectively (Table 1). From 1 μg of protein, the number of OMV particles from normoxia and hypoxia was slightly different with 2.71 × 10^8^ and 2.19 × 10^8^ particles, respectively. Obviously, from an equivalent volume of culture supernatant (4.8 L), the protein yield and total particle number of hAbOMVs were approximately 3 times higher than those of nAbOMVs (Table 1 and S1 Dataset).

**Table 1:** Total protein, particle diameter and particle number of nAbOMVs and hAbOMVs obtained from 4.8 L of *A*. *baumannii* ATCC19606 culture supernatants (n = 3).

	Total protein (mg) ± SD	Particle diameter (nm) ± SD	Particle number (×10^8^per 1 μg of protein) ± SD	Total particle number (×10^11^) ± SD
**nAbOMVs**	0.62 ± 0.12	187 ± 7.32	2.72 ± 0.25	1.68 ± 0.35
**hAbOMVs**	2.12 ± 0.30	189 ± 20.53	2.18 ± 0.10	4.62 ± 0.70

### Differential proteomic analysis between nAbOMVs and hAbOMVs

To investigate the differences in protein profiles between nAbOMVs and hAbOMVs, quantitative proteome analysis was performed. A total of 584 proteins were identified from the triplicated preparations of nAbOMVs and hAbOMVs. The differential comparisons between nAbOMVs and hAbOMVs indicated that 30 proteins were significantly higher in nAbOMVs (*P* < 0.05) (Table 2). Among these, six proteins are hypothetical proteins with unknown function. Alkyl hydroperoxide reductase C was detected only in nAbOMVs. The proteins enriched more than 10-fold in nAbOMVs were polysaccharide biosynthesis tyrosine autokinase, dihydrolipoamide acetyltransferase component of pyruvate dehydrogenase complex, outer-membrane lipoprotein carrier protein, and polysaccharide biosynthesis export family protein. The proportion of cytosolic, cytoplasmic membrane, periplasmic. outer membrane, extracellular and unknown location proteins was 7:6:3:2:1:11. The proteins that have been shown to associate with *A*. *baumannii* virulence were outer membrane receptor FepA, polysaccharide biosynthesis tyrosine autokinase and taurine ABC transporter substrate-binding protein [24].

**Table 2:** List of proteins enriched in nAbOMVs with *P* value < 0.05.

Gene name	Protein	Predicted MW (kDa)	Predicted location*	Fold- change	*P*-value
*ahpC*	Alkyl hydroperoxide reductase C	20.8	C	Infinity^†^	0.020
*ptk*	Polysaccharide biosynthesis tyrosine autokinase	81.6	Cym	27.24	<0.001
*aceF*	Dihydrolipoamide acetyltransferase component of pyruvate dehydrogenase complex	68.4	C	23.19	0.020
*lolA*	Outer-membrane lipoprotein carrier protein	25.3	P	10.46	<0.001
*wza*	Polysaccharide biosynthesis export family protein	40.6	O	10.18	0.019
*icmF*	Type VI secretion protein	143.9	Cym	9.28	0.049
HMPREF0010_02682	Hypothetical protein	24.4	Unk	9.02	0.004
DOL94_08760	Protein CsuA	19.7	Unk	8.49	0.017
HMPREF0010_02860	SPFH/Band 7/PHB domain protein	31.0	Unk	7.26	<0.001
HMPREF0010_02513	Penicillin-binding protein 1A	94.8	E	5.18	0.033
*ggt*	Glutathione hydrolase proenzyme	69.8	P	4.77	0.047
*fadA*	Acetyl-CoA C-acyltransferase FadA	41.1	C	4.20	0.009
*acrA*	Acriflavine resistance protein A/MULTISPECIES	43.8	Unk	4.12	0.001
*cydA*	Cytochrome bd oxidase subunit I	58.7	Cym	4.11	0.048
HMPREF0010_02797	Sel1 repeat protein	29.6	Unk	3.96	0.019
ATCC19606_33910	LprI domain-containing protein	14.2	Unk	3.95	0.049
*queF*	NADPH-dependent 7-cyano-7-deazaguanine reductase QueF	31.0	C	3.91	0.030
*mqo*	Malate dehydrogenase	60.4	C	3.35	0.005
HMPREF0010_00307	Hypothetical protein	19.1	Unk	3.35	0.004
*tauA*	Taurine ABC transporter substrate-binding protein	38.0	P	2.92	0.006
HMPREF0010_02833	Hypothetical protein	18.8	Unk	2.88	0.041
*tolA*	Gramicidin S synthase	50.6	Unk	2.75	0.030
*atpB*	ATP synthase subunit A	32.4	Cym	2.70	0.025
ATCC19606_13250	RDD family protein	28.5	Cym	2.62	0.027
ATCC19606_04350	Hypothetical protein	38.6	Unk	2.57	0.004
*hlyD*	RND transporter	40.7	Cym	2.09	0.050
HMPREF0010_02162	Hypothetical protein	17.7	Unk	1.95	0.028
A7M90_10095	Hypothetical protein	46.6	C	1.78	0.040
*dnaK*	Chaperone protein DnaK	69.4	C	1.77	0.006
HMPREF0010_01517	Outer membrane receptor FepA (TonB-dependent siderophore receptor)	82.8	O	1.60	0.045

*C: Cytosolic; Cym: Cytoplasmic membrane; P: Periplasmic; O: Outer membrane; E: Extracellular; Unk: Unknown (the location is unpredictable).

^†^Fold change was calculated based on 5197.7 counts for nAbOMVs and 0 count for hAbOMVs.

The proteins enriched in hAbOMVs with *P* value < 0.05 are shown in Table 3. Of these 25 proteins, two are hypothetical proteins with unknown function and several proteins are enzymes involving in metabolism. GntR family transcriptional regulator was detected only in hAbOMVs. The proteins enriched more than 10-fold in hAbOMVs were haloacid dehalogenase (HAD) hydrolase, family IB and methionine aminopeptidase. The proportion of cytosolic, cytoplasmic membrane, periplasmic. outer membrane, extracellular and unknown location proteins was 14:5:0:0:0:6. Among these proteins, outer-membrane lipoprotein LolB, and high-affinity zinc transporter ATPase are lipoproteins that have been shown to associate with *A*. *baumannii* virulence [24].

**Table 3:** List of proteins enriched in hAbOMVs with *P* value < 0.05.

Gene name	Protein	Predicted MW (kDa)	Predicted location*	Fold- change	*P*-value
HMPREF0010_01056	GntR family transcriptional regulator	24.8	C	Infinity^†^	0.020
HMPREF0010_02207	HAD hydrolase, family IB	25.0	C	21.81	<0.001
*map*	Methionine aminopeptidase	30.4	C	19.84	0.035
*gtr6*	Glycosyl transferase	45.1	C	9.81	0.048
*ftsI*	Peptidoglycan D, D-transpeptidase FtsI	67.7	Cym	8.67	0.041
HMPREF0010_01031	IclR helix-turn-helix domain protein	32.0	C	8.49	0.049
*lolB*	Outer-membrane lipoprotein LolB	21.1	Unk	8.16	0.036
*bioA*	Adenosylmethionine-8-amino-7-oxononanoate aminotransferase	47.8	C	6.70	0.007
*accD*	Acetyl-coenzyme A carboxylase carboxyl transferase subunit beta	33.0	C	6.42	0.012
*rpsS*	30S ribosomal protein S19	10.2	C	5.90	0.004
HMPREF0010_03595	DNA helicase	55.3	C	5.50	0.034
*pyrG*	CTP synthase	61.0	C	4.41	0.022
HMPREF0010_00687	YbaB/EbfC family nucleoid-associated protein	12.0	Unk	4.31	0.011
*pyrD*	Dihydroorotate dehydrogenase	36.0	Cym	3.76	0.023
*rpsT*	30S ribosomal protein S20	9.7	C	3.74	0.044
B9X95_03935	DUF799 domain-containing protein	24.1	Unk	3.72	0.007
HMPREF0010_01246	Ribonucleotide-diphosphate reductase subunit beta	48.9	C	3.50	0.039
*hslR*	Heat shock protein 15	17.1	C	3.38	0.004
*rpmB*	50S ribosomal protein L28	9.1	C	3.30	0.012
*znuC*	High-affinity zinc transporter ATPase	29.5	Cym	3.10	0.022
HMPREF0010_00917	Hypothetical protein	35.0	Unk	3.07	0.001
ATCC19606_09150	DUF1615 domain-containing protein	45.8	Unk	2.51	0.020
ATCC19606_25890	Hypothetical protein	13.1	Unk	2.38	0.016
HMPREF0010_02256	Phosphate ABC transporter, phosphate-binding protein	37.0	Cym	1.91	0.031
*metQ_1*	MetQ/NlpA family ABC transporter substrate-binding protein	31.3	Cym	1.42	0.003
*omp38*	Outer membrane protein OmpA^‡^	38.4	O	2.20	0.252

*C: Cytosolic; Cym: Cytoplasmic membrane; P: Periplasmic; O: Outer membrane; E: Extracellular; Unk: Unknown (the location is unpredictable).

^†^Fold change was calculated based on 13123.0 counts for hAbOMVs and 0 count for nAbOMVs.

^‡^OmpA, a major virulence factor of *A*. *baumannii*, was detected higher in hAbOMVs with 2.20-fold change but statistical analysis did not show significant difference (*P* = 0.252).

Since OmpA is a key virulence factor of *A*. *baumannii*, the amount of OmpA in nAbOMVs and hAbOMVs was also focused. As shown in Table 3, the amount of OmpA was 2.2-fold higher in hAbOMVs than that in nAbOMVs, but the statistical analysis shows no significant difference (*P* = 0.252).

These proteomic data demonstrated that the types and amounts of several proteins involved in virulence of *A*. *baumannii* differed between nAbOMVs and hAbOMVs. Therefore, we further examined the difference in the effects of nAbOMVs and hAbOMVs on host immune response, cytotoxicity, and bacterial infections.

### Effects of nAbOMVs and hAbOMVs on host immune responses

**Fig 2:**
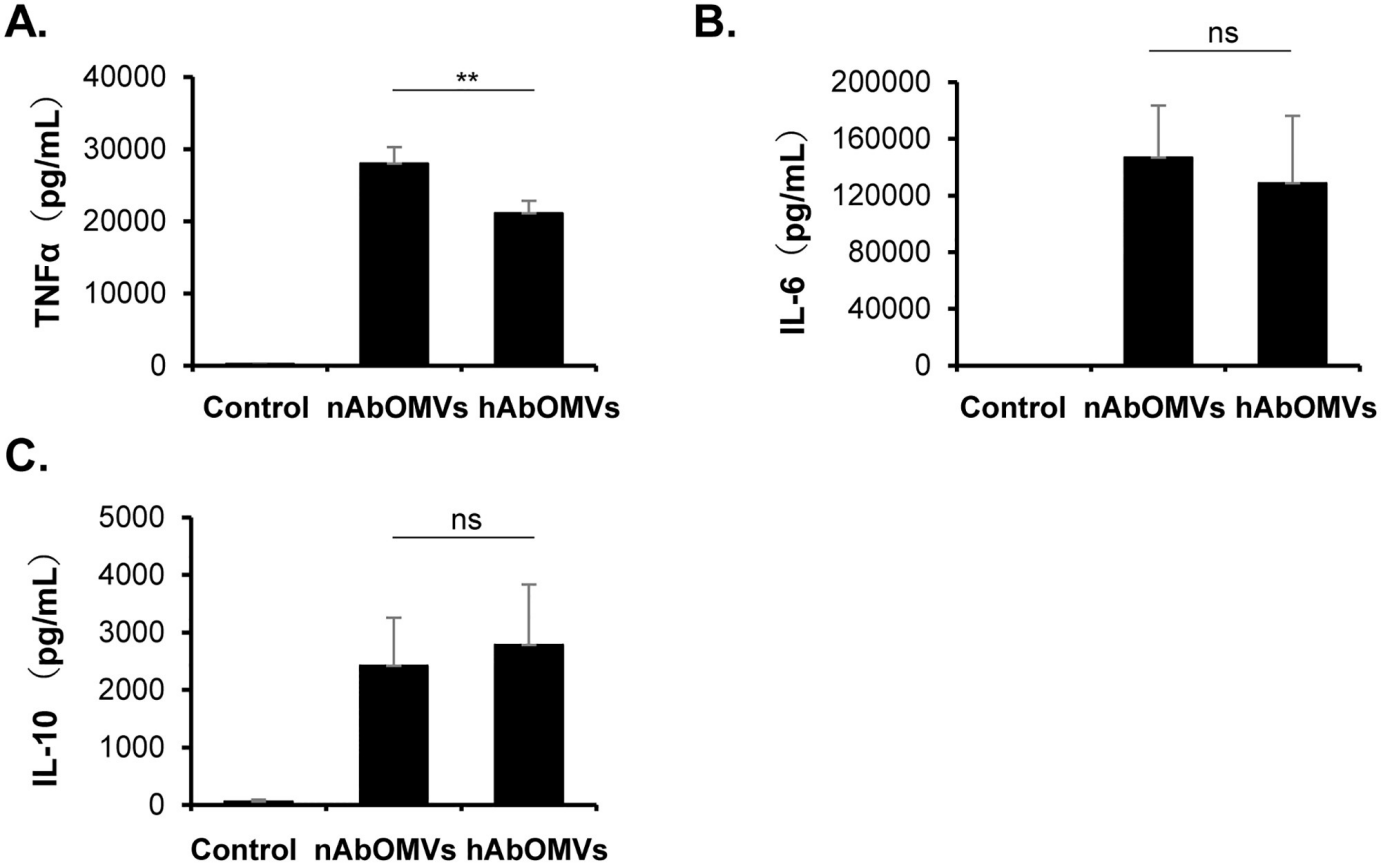
Cytokine production from mouse macrophages after stimulation with nAbOMVs and hAbOMVs. RAW264.7 cells (1 × 10^6^ cells/mL/well) were incubated with 12.5 μg/mL AbOMVs for 48 h at 37°C. The production of (A) TNF-α, (B) IL-6, and (C) IL-10 in the culture supernatant was determined by ELISAs (n = 6 from 2 independent experiments). *P*-value was calculated using Mann-Whitney U-test. Asterisk (**) indicates significant difference between nAbOMVs and hAbOMVs (*P* < 0.01). ns: not significant.

To examine the different effects of nAbOMVs and hAbOMVs on host immune responses, mouse macrophage RAW264.7 cells were prepared and stimulated with 12.5 μg/mL of each AbOMVs. At 48 h of stimulation, cytokines in the culture supernatant were measured by ELISAs. As shown in Fig 2, the purified AbOMVs from both normoxia and hypoxia significantly stimulated the production of TNF-α, IL-6, and IL-10 from RAW264.7 cells compared to non-AbOMV control. These results suggest that AbOMVs from both conditions strongly stimulate host immune responses. In addition, TNF-α production by nAbOMV stimulation was significantly higher than that by hAbOMVs (Fig 2A and S2 Dataset). However, this different effect was not observed in IL-6 and IL-10 production (Fig 2B and 2C, and S2 Dataset).

### Cytotoxic effects of nAbOMVs and hAbOMVs to human lung epithelial cells

**Fig 3:**
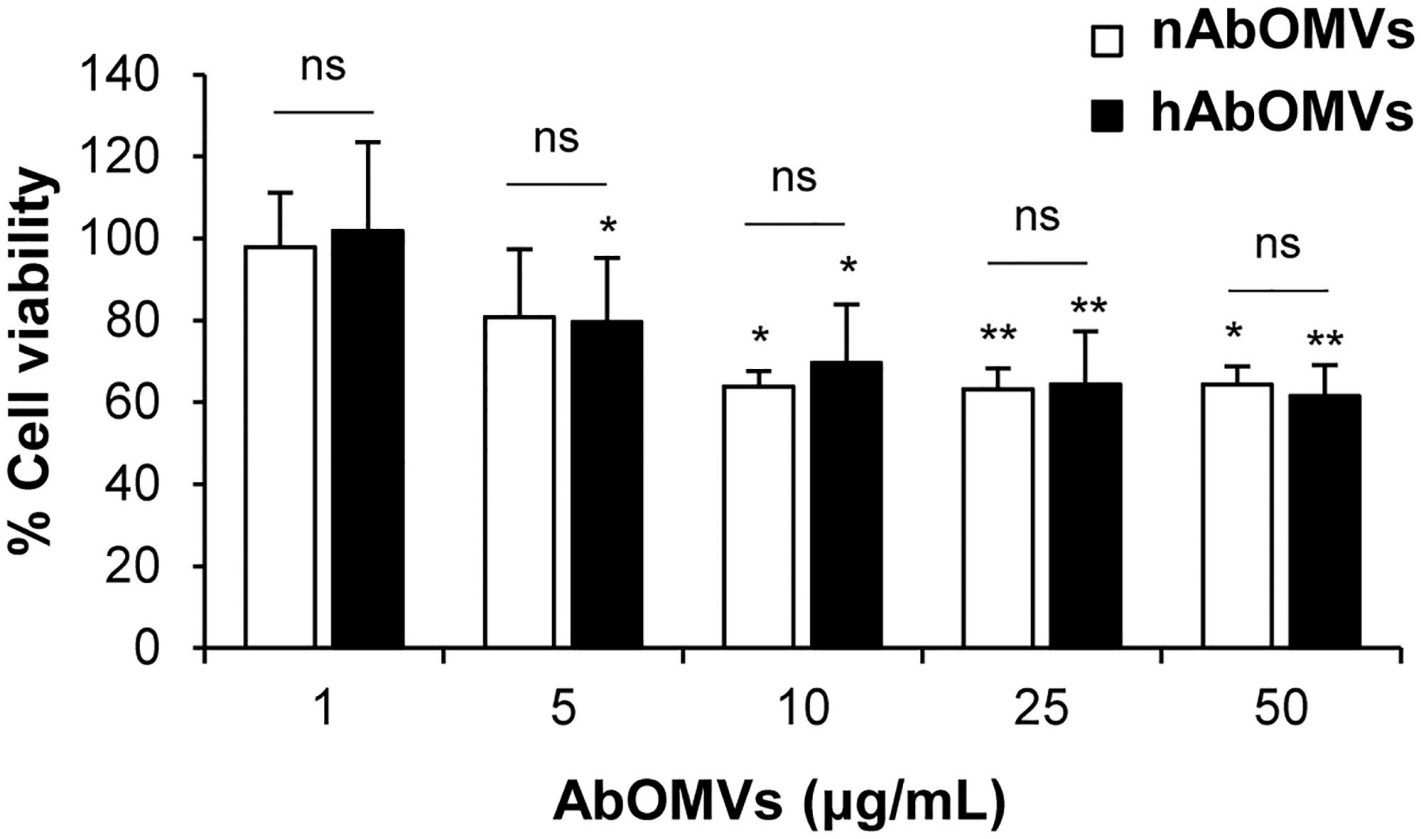
Cytotoxic effect of nAbOMVs and hAbOMVs to lung epithelial cells. A549 cells (1 × 10^4^ cells/100 μL/well) were seeded in 96-well plate and incubated with AbOMVs at 37°C under 5%CO_2_. At 12 h of incubation, 10 μL of WST-1 reagent was added into each well and the absorbance was measured at 450 nm. Cell viability in the reactions without AbOMVs (control) was calculated as 100% (n = 7 from 3 independent experiments). *P*-value was calculated using Kruskal Wallis H-test with post hoc Mann-Whitney U-test. Asterisks indicates significant difference to non-AbOMV control (*: *P* < 0.05, **: *P* < 0.01). *P*-value of difference between nAbOMVs and hAbOMVs was calculated using Mann-Whitney U-test. ns: not significant.

The cytotoxic effects of nAbOMVs and hAbOMVs to A549 lung epithelial cells were examined. At 12 h after incubation of A549 cells with AbOMVs, WST-1 reagent was added, and the absorbance at 450 nm was measured. The cytotoxic effects of nAbOMVs and hAbOMVs to A549 cells are shown in Fig 3 and S3 Dataset. In the presence of AbOMVs, the cell viability was decreased compared to non-AbOMV control. The decrease in cell viability was dose-dependent up to a concentration of 10 μg/mL. At AbOMVs concentrations above 10 μg/mL, the cell viability remained constant at approximately 63%. nAbOMVs and hAbOMVs reduced cell viability with a similar trend with no significant difference.

### Effects of nAbOMVs and hAbOMVs on bacterial infections into A549 cells

**Fig 4:**
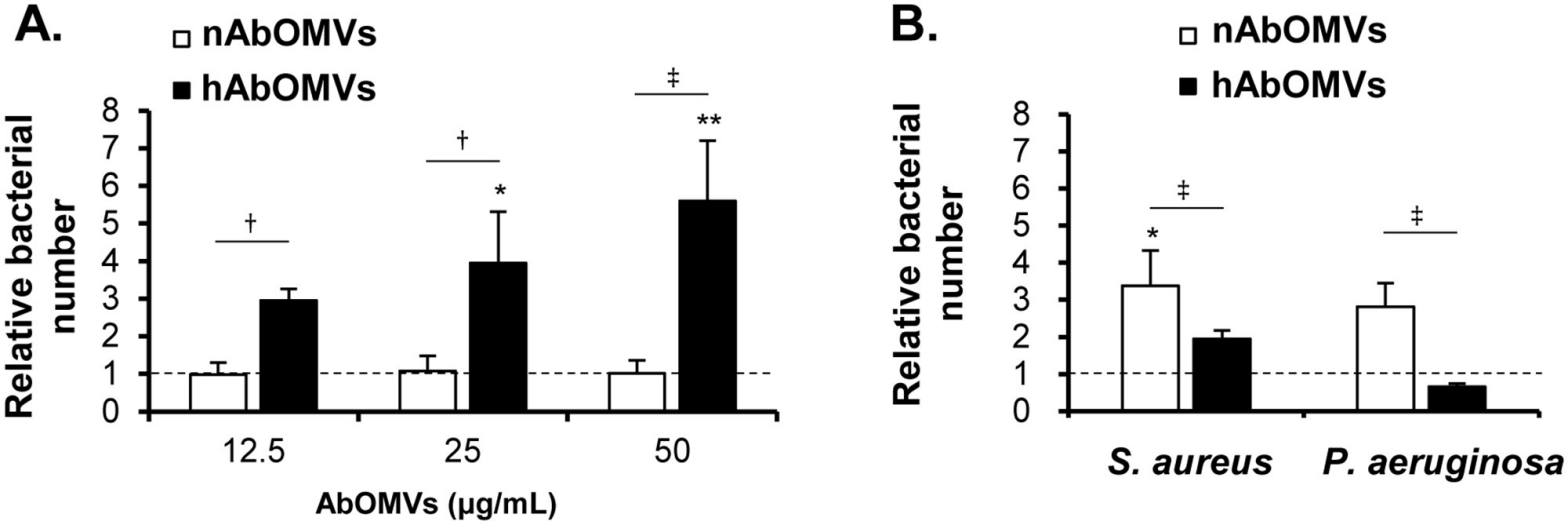
Effect of nAbOMVs and hAbOMVs on bacterial infections into lung epithelial cells. A549 cells (5 × 10^4^ cells/mL/well) were seeded in a 24-well plate. (A) A549 cells were infected with *A*. *baumannii* at MOI = 50 in the presence of AbOMVs for 5 h (n = 6 from 2 independent experiments). Bacterial number from the condition without AbOMVs is referred to 1.0 (dotted line). (B) A549 cells were infected with *S*. *aureus* or *P*. *aeruginosa* at MOI = 50 in the presence of 12.5 μg/mL AbOMVs for 2 h (n = 6 from 2 independent experiments). Invasion efficacy was expressed as the relative number to the condition without AbOMVs, which is referred to 1.0 (dotted line). *P*-value of difference to non-AbOMV control was calculated using Kruskal Wallis H-test with post hoc Mann-Whitney U-test. *: *P* < 0.05, **: *P* < 0.01. *P*-value of difference between nAbOMVs and hAbOMVs was calculated using Mann-Whitney U-test. †: *P* < 0.05, ‡: *P* < 0.01.

The effect of nAbOMVs and hAbOMVs on *A*. *baumannii* infection into the A549 cells was examined. A549 cells were infected with *A*. *baumannii* (MOI = 50) in the presence of AbOMVs for 5 h, then the invading *A*. *baumannii* was enumerated. The results in Fig 4A and S4 Dataset are expressed as relative numbers to the invading *A*. *baumannii* without AbOMVs (set at 1.0). At various concentrations of nAbOMVs, the numbers of *A*. *baumannii* invading into A549 cells were constant and comparable to that of non-AbOMV control. In contrast, hAbOMVs promoted the number of *A*. *baumannii* invading into A549 cells in a dose-dependent manner. The invading numbers of *A*. *baumannii* in the presence of 12.5–50 μg/mL nAbOMVs and hAbOMVs were significantly different.

The effects of AbOMVs on infections of the common coexisting pathogens, *S*. *aureus* and *P*. *aeruginosa*, were also investigated. A549 cells were infected with *S*. *aureus* or *P*. *aeruginosa* (MOI = 50) in the presence of 12.5 mg/mL of nAbOMVs or hAbOMVs for 2 h, and the invading bacterial cells were then enumerated. The results are expressed as relative numbers to the invading *S*. *aureus* or *P*. *aeruginosa* without AbOMVs (set at 1.0). As shown in Fig 4B and S4 Dataset, nAbOMVs promoted the invasion of both *S*. *aureus* and *P*. *aeruginosa* into A549 cells compared to non-AbOMV control (referred to 1.0). On the other hand, hAbOMVs enhanced the invading number of *S*. *aureus* but not *P*. *aeruginosa*. Overall infection results showed that nAbOMVs significantly promoted invasion of *S*. *aureus* and *P*. *aeruginosa* more than hAbOMVs. This was inconsistent with their effects on *A*. *baumannii* infection (Fig 4A).

## Discussion

*A*. *baumannii* is a major causative agent of nosocomial infections [2]. At infected sites such as skin and lungs, this microorganism often co-infects with *S*. *aureus* and *P*. *aeruginosa*, which results in severe prognosis [25, 26]. It has been known that the extracellular membrane vesicles released from bacteria play an important role in bacterial pathogenicity. Based on structure and composition, these vesicles can deliver the virulence factors directly to the host cells and transmit signal information to other pathogens for promoting virulence [14, 27]. The composition and virulence of AbOMVs prepared under aerobic condition, which is suitable for *A*. *baumannii* growth, have been reported [28]. However, it has been reported that infected tissues are hypoxic because of increased oxygen consumption due to activation of immune-related cells and decreased oxygen supply due to damage to blood vessels. Thus, while normal lung tissue is in contact with the atmosphere and have a high oxygen concentration, the infected area is thought to be hypoxia [16]. Under this hypoxic stress, bacteria need to alter their gene expression profiles to survive, especially the proteins related to nutrient transport and metabolism [29]. It has been demonstrated that the amount of OMVs released from *P*. *aeruginosa* is altered under hypoxia [17]. Therefore, we hypothesized that the amount and protein profile of AbOMVs prepared under hypoxia would differ from those under normoxia. We also expected that the difference in the protein profiles in the AbOMVs would alter their role in virulence. Therefore, in the present study, AbOMVs under normoxic and hypoxic conditions were purified and their different effects on host immune response, cytotoxicity and bacterial infections were investigated.

As shown in Table 1, there was no significant difference in the particle size and particle concentration (per 1 μg protein) between the purified nAbOMVs and hAbOMVs. These particle sizes were comparable to that of *A*. *baumannii* OMVs isolated by Sun Li, et al. [15]. However, the protein yield as well as the total particle number of hAbOMVs were approximately 3-fold higher than those of nAbOMVs. These obtaining yields were constant from triplicated preparations. Together with the differences in growth of *A*. *baumannii* under normoxic and hypoxic conditions, our results indicate that the hypoxia, a stress condition for *A*. *baumannii*, remarkably promotes AbOMVs production over normoxic condition.

Differential proteomic analysis was performed to observe differences in the protein profiles of nAbOMVs and hAbOMVs. As shown in Tables 2 and 3, 30 proteins were significantly enriched in nAbOMVs and 25 proteins were significantly enriched in hAbOMVs. The enriched proteins in nAbOMVs that have been shown to play a role in *A*. *baumannii* virulence are the outer membrane receptor FepA (TonB-dependent siderophore receptor), polysaccharide biosynthesis tyrosine autokinase and taurine ABC transporter substrate-binding protein. FepA has been shown to be involved in iron-scavenging system and promote the growth of *A*. *baumannii* [30, 31]. The tyrosine autokinase is markly elevated to export the exopolysaccharide during biofilm formation [32], and the taurine ABC transporter substrate-binding protein is involved in the transport of taurine as a sulfur source and in competition for taurine utilization between the nosocomial pathogens and host [33].

Proteins enriched in hAbOMVs that have been shown to be involved in the virulence of *A*. *baumannii* include outer-membrane lipoprotein LolB and high-affinity zinc transporter ATPase. The outer-membrane lipoprotein LolB constitutes the Lol lipoprotein transport system and transports lipoproteins to the outer membrane in especially LPS-deficient *A*. *baumannii* [34]. The high-affinity zinc transporter ATPase ZnuC constitutes the ZnuABC transporter to acquire zinc within the hostile environment of the host [35].

In addition to these known proteins, the hypothetical proteins with unknown function might also contribute to the virulence of AbOMVs. Moreover, OMVs from Gram-negative bacteria are known to carry LPS, peptidoglycan as well as lipoproteins to induce inflammatory response via pattern recognition receptor-mediated interactions [36, 37]. Therefore, we further compared the cytokine responses from mouse macrophages after nAbOMVs and hAbOMVs stimulations. As shown in Fig 2, the production of TNF-α, IL-6 and IL-10 from RAW264.7 cells was significantly increased after stimulations with nAbOMVs and hAbOMVs. Although there was no significant difference in the production of IL-6 and IL-10 between nAbOMVs and hAbOMVs stimulation, the TNF-α production caused by nAbOMVs stimulation was significantly higher than that by hAbOMVs. A previous study has suggested that OmpA in OMVs is a key factor for cytokine stimulation because the OMVs from ΔOmpA mutant of *A*. *baumannii* induce lower levels of TNF-α and IL-6 compared to OMVs of the wild-type strain [14]. Although our proteomic analysis revealed that OmpA seems to be enriched in hAbOMVs for (2.20-fold change), there was no statistically significant difference (*P* value = 0.252). In contrast, the proportion of polysaccharide biosynthesis-related proteins and the periplasmic proteins was enriched in nAbOMVs. Thus, it is possible that polysaccharides and peptidoglycans in periplasm may be enriched in nAbOMVs, and these molecules may contribute to the high titer of TNF-α due to nAbOMVs stimulation.

It has been shown that AbOMVs exhibit the cytotoxic effect to the host cells [38], and a key toxic factor is OmpA because Skerniškytė et al. demonstrated that the cytotoxicity of AbOMVs from *A*. *baumannii* lacking OmpA decreased [14]. Since our proteomic analysis revealed that the amount of OmpA in nAbOMVs and hAbOMVs was not significantly different (*P* value = 0.252), it was not surprising that the cytotoxic effect of nAbOMVs and hAbOMVs to human lung epithelial cells was comparable (Fig 3). Interestingly, the cytotoxic effect of both AbOMVs was not increased at the concentration above 10 μg/mL. Further experiments are required to explain the details of this mechanism.

The effect of nAbOMVs and hAbOMVs on bacterial infection was also examined to determine whether nAbOMVs and hAbOMVs promote *A*. *baumannii* invasion. It has been shown that OmpA is required for *A*. *baumannii* to adhere to and invade the epithelial cells [12]. The number of *ΔompA A*. *baumannii* infections into epithelial cells significantly decreased compared to the wild type, and pretreatment of recombinant OmpA to the host cells inhibits the interaction of *A*. *baumannii* with the epithelial cells [12]. Since OmpA was comparably detected in both nAbOMVs and hAbOMVs, we expected that both AbOMVs may inhibit *A*. *baumannii* internalization. Surprisingly, our results showed that nAbOMVs did not inhibit *A*. *baumannii* invasion even the concentration was up to 50 μg/mL. In contrast, hAbOMVs significantly enhanced *A*. *baumannii* invasiveness in a dose-dependent manner (Fig 4A). These results suggest that some proteins enriched in hAbOMVs (in Table 3) may act as effectors to regulate cytoskeleton dynamics and induce *A*. *baumannii* uptake into lung epithelial cells. In particular, under stressful hypoxic condition, *A*. *baumannii* may highly release these effectors together with OMVs to promote its infectivity.

*A*. *baumannii* often causes infections of the skin and lungs where *P*. *aeruginosa* and *S*. *aureus* usually coexist. We hypothesized that there may be AbOMV-mediated interactions between these bacteria. Therefore, the effect of AbOMVs on *S*. *aureus* and *P*. *aeruginosa* infection was also examined. In contrast to the effects of AbOMVs on *A*. *baumannii* infection, nAbOMVs promoted *S*. *aureus* and *P*. *aeruginosa* invasion into A549 epithelial cells. Although hAbOMVs also promoted *S*. *aureus* invasion, these vesicles did not affect *P*. *aeruginosa* invasiveness. The invasion mechanism of *S*. *aureus* into the epithelial cells is suggested to be mediated by fibronectin that forms bridging between host cell and *S*. *aureus* surface [39]. In addition, the invasion mechanism of *P*. *aeruginosa* is mediated by the formation of bacterial aggregates on the surface of epithelial cells [40]. Since these mechanisms are different from that of *A*. *baumannii* which invades into the epithelial cells via a zipper-like mechanism [12, 41], the different effectors in nAbOMVs and hAbOMVs may reflect their different effects on these bacterial infections. During infection, *A*. *baumannii* probably competes other bacterial species [42]. In particular, under stress of hypoxic condition, *A*. *baumannii* may have highly competitive with *P*. *aeruginosa* because both of them preferentially use aerobic respiration [43, 44]. Therefore, a high hAbOMVs production with the effective molecules from hypoxic condition would be a beneficial strategy for *A*. *baumannii* to promote its own survival and outcompete *P*. *aeruginosa*. It is important to noted that OMVs do not contain only proteins or lipoproteins. The other bioactive molecules such as LPS, peptidoglycan, DNA and RNA in OMVs are also possible to contribute to these effects. Although further investigation of the molecular mechanisms of AbOMVs involved in bacterial infection into the host cells is required, we expected that the proteins reported in this study would provide great benefits for progression in the research field of OMVs and their relationship to infection. Taken all together, our results indicated AbOMVs from normoxia and hypoxia exhibited different effects to promote the infections of *A*. *baumannii* and other bacteria.

## Conclusion

This study demonstrated that *A*. *baumannii* under hypoxic condition produces higher amount of OMVs than that under normoxic condition. The protein profile and the pathogenic effect of AbOMVs under normoxic and hypoxic are also different. Although the cytotoxic effect of nAbOMVs and hAbOMVs to human lung epithelial cells was comparable, the OMVs released from *A*. *baumannii* under normoxia promoted higher TNF-α production and enhanced *S*. *aureus* and *P*. *aeruginosa* internalization into lung epithelial cells. On the other hand, OMVs release from *A*. *baumannii* under hypoxia promoted invasion of *A*. *baumannii* into lung epithelial cells. This finding provides new insight into the complex interactions between infecting pathogens and host mediated by OMVs, in particular the different effect under normoxic and hypoxic conditions.

## Supporting information

S1 Dataset Total protein, particle diameter and particle number of nAbOMVs and hAbOMVs obtained from 4.8 L of *A*. *baumannii* ATCC19606 culture supernatants. (XLSX)

S2 Dataset Cytokine production from mouse macrophages after stimulation with nAbOMVs and hAbOMVs. (XLSX)

S3 Dataset Cytotoxic effect of nAbOMVs and hAbOMVs to lung epithelial cells. (XLSX)

S4 Dataset Effect of nAbOMVs and hAbOMVs on bacterial infections into lung epithelial cells. (XLSX)
